# Synergistic Cellulose Hydrolysis Dominated by a Multi-Modular Processive Endoglucanase from *Clostridium cellulosi*

**DOI:** 10.3389/fmicb.2016.00932

**Published:** 2016-06-15

**Authors:** Min Yang, Kun-Di Zhang, Pei-Yu Zhang, Xia Zhou, Xiao-Qing Ma, Fu-Li Li

**Affiliations:** ^1^College of Environmental Science and Engineering, Qingdao UniversityQingdao, China; ^2^Shandong Provincial Key Laboratory of Energy Genetics, Key Laboratory of Biofuel, Qingdao Institute of BioEnergy and Bioprocess Technology, Chinese Academy of SciencesQingdao, China; ^3^Exploration & Production Research Institute, China Petroleum & Chemical CorporationBeijing, China

**Keywords:** cellulose, secretome, synergism, processive endoglucanase, β-glucosidase

## Abstract

Recalcitrance of biomass feedstock remains a challenge for microbial conversion of lignocellulose into biofuel and biochemicals. *Clostridium cellulosi*, one thermophilic bacterial strain dominated in compost, could hydrolyze lignocellulose at elevated temperature by secreting more than 38 glycoside hydrolases belong to 15 different families. Though one multi-modular endoglucanase *Cc*Cel9A has been identified from *C. cellulosi* CS-4-4, mechanism of synergistic degradation of cellulose by various cellulases from strain CS-4-4 remains elusive. In this study, *Cc*Cel9A, *Cc*Cel9B, and *Cc*Cel48A were characterized as processive endoglucanase, non-processive endoglucanase, and exoglucanase, respectively. To understand how they cooperate with each other, we estimated the approximate concentration ratio on the zymogram and optimized it using purified enzymes *in vitro*. Synergism between individual glycoside hydrolase during cellulose hydrolysis in the mixture was observed. *Cc*Cel9A and *Cc*Cel48A could degrade cellulose chain from non-reducing ends and reducing ends, respectively, to cello-oligosaccharide. *Cc*Cel9B could cut cellulose chain randomly and cello-oligosaccharides with varied length were released. In addition, a β-glucosidase BlgA from *Caldicellulosiruptor* sp. F32 which could cleave cello-oligosaccharides including G2-G6 to glucose was added to the enzyme mixture to remove the product inhibition of its partners. The combination and ratios of these cellulases were optimized based on the release rate of glucose. Hydrolysis of corn stalk was conducted by a four-component cocktail (*Cc*Cel9A:*Cc*Cel9B:*Cc*Cel48A:BlgA = 25:25:10:18), and only glucose was detected as main production by using high-performance anion-exchange chromatography. Processive endoglucanase *Cc*Cel9A, dominated in secretome of *C. cellulosi*, showed good potential in developing cellulase cocktail due to its exquisite cooperation with various cellulases.

## Introduction

Lignocellulose is the most abundant renewable biomass for the production of biofuels and biochemicals. The bioconversion of lignocellulose to fuel and chemical is carried out via three processes: pretreatment, enzymatic hydrolysis, and fermentation (Lynd et al., 2005). Due to the recalcitrance and complexity of lignocellulose, the cost of enzymatic hydrolysis is very high, which is the bottleneck of this industry. Therefore, much effort has been made to improve the enzymatic hydrolysis efficiency, mainly focusing on reducing the enzyme amount and hydrolysis time. Development of cellulose cocktails became a mainstream strategy to enhance the process. The components of the cocktails could cooperate with each other in modifying the structure of crystalline cellulose, thus making cellulose more accessible for cellulases (Jeoh et al., 2006; Arantes and Saddler, 2010). Therefore, mining cellulases candidates which can synergize in nature was imperative.

*Clostridium cellulosi* is an obligately anaerobic, thermophilic Gram-positive bacterium (He et al., 1991). Although it appears not to produce cellulosome, *C. cellulosi* utilizes a broad range of carbon sources such as fructose, glycogen, inulin, mannitol, starch, trehalose, and xylose for generation of hydrogen, carbon dioxide, ethanol and acetic acid, which was more than *C. thermocellum* does. In the genomic sequence of *C. cellulosi* CS-4-4 a gene cluster containing five open reading frames including Ccel_3879 encoding an exoglucanase, Ccel_3880 encoding an endoglucanase, Ccel_3881 encoding a putative glycoside hydrolase, Ccel_3882 encoding a xylanase, and Ccel_3883 encoding another putative glycoside hydrolase was identified. LC-MS analysis of the bands on zymogram of spent culture from *C. cellulosi* CS-4-4 indicated that Ccel_3880 and Ccel_3883 played the key roles in hydrolyzing xylan and CMC (carboxymethylcellulose). Ccel_3879, as the only band appearing on the exoglucanase zymogram, was the most abundant exoglucanase in the secretome of *C. cellulosi* CS-4-4. Conserved domain analysis of the three ORFs showed that both Ccel_3880 and Ccel_3883 were family 9 glycoside hydrolases (*Cc*Cel9A and *Cc*Cel9B), while Ccel_3879 belonged to family 48 glycoside hydrolase (*Cc*Cel48A). Additionally, *Cc*Cel9A possessed one GH module and five CBMs (carbohydrate-binding module), which is scarce in Prokaryotes (see ESM_2.pdf in Supplementary Material) (Zhang et al., 2014).

Cellulose systems exhibit higher activity than the sum of the activities of individual enzymes, which is known as synergism. Four types of synergism have been proposed: (i) endo-exo synergy between endoglucanases and exoglucanases, (ii) exo-exo synergy between reducing-end exoglucanases and non-reducing-end exoglucanases, (iii) synergy between exoglucanases, and β-glucosidases that remove cellobiose and cellodextrins as repressive end-products, and (iv) intramolecular synergy between catalytic modules and CBMs (Din et al., 1994; Teeri, 1997). The synergism between GH family 9 processive endoglucanase and GH family 48 exoglucanase has been proved in *C. phytofermentans* (Zhang et al., 2010) and *Thermobifida fusca* (Kostylev and Wilson, 2014). In *T. fusca* a model of synergistic cooperation between *Tf* Cel9A and *Tf* Cel48A has been proposed. *Tf* Cel9A provides accessible substrate for *Tf* Cel48A. *Tf* Cel48A preferentially hydrolyzes looser chains, replenishing the smooth surface for *Tf* Cel9A. *Tf* Cel9A is the first identified processive endoglucanase, which can cleave cellotetrose from the non-reducing end of a cellulose chain processively and hydrolyze cellotetrose to glucose, cellobiose, and cellotriose sequentially (Sakon et al., 1997). Thereafter, several processive endoglucanases, which possess both endo- and exoglucanases activities as *Tf* Cel9A, have been discovered and characterized (Riedel and Bronnenmeier, 1999; Mandelman et al., 2003; Zverlov et al., 2003, 2005; Berger et al., 2007; Zhang et al., 2010; Jeon et al., 2012). However, how the processive endoglucanase, non-processive endoglucanase, and exoglucanase cooperate with each other in the hydrolyzing process remains unknown.

In this study, we detected the cellulose hydrolyzing activities of a series of the mixture enzymes with various ratios to evaluate the cooperation between *Cc*Cel9A, *Cc*Cel9B, and *Cc*Cel48A. Furthermore, we added a β-glucosidase BlgA from *Caldicellulosiruptor* sp. F32 to the mixtures (Meng et al., 2015) to improve the effect of cellulose saccharification by hydrolyzing all cello-oligosaccharides to glucose, which will be beneficial for utilization by engineering strains in industrial production.

## Materials and methods

### Strains and chemicals

The *Escherichia coli* strains Trans1-T1 and BL21(DE3) used in plasmids construction were purchased from TransGen Biotech (Beijing, China). CMC was from Sigma-Aldrich (St. Louis, USA) and Whatman no. 1 filter paper was from GE Healthcare (Stockholm, Sweden). Cello-oligosaccharides including cellobiose (G2), cellotriose (G3), cellotetraose (G4), cellopentaose (G5), and cellohexose (G6) were all from Megazyme (Wicklow, Ireland). All the reagents were of analytical grade.

### Plasmid construction

The gene of *Cc*Cel48A was amplified from the genomic DNA of *C. cellulosi* CS-4-4 (GenBank accession Number: KF434246) using a forward primer (5′-GCGGATGATGGGACCTAC AAGGCGAAG-3′) and a reverse primer (5′- CGGTTCAACTCC CCAAATTAGCTCGTCGC-3′). The polymerase chain reaction (PCR) products were cloned into *p*EASY-E2 vector and transformed into *E. coli* Trans1-T1. After overnight cultivation, several clones were selected for verification by colony PCR and the DNA insert was sequenced by Sunny Bio-technology Co. Ltd. (Shanghai, China) to confirm the integrity of the gene. Then the expression vector for *Cc*Cel48A was extracted and transformed into *E. coli* BL21(DE3). Afterwards, a single positive clone was selected and cultivated to make competent cells, which was as a host to accept the chaperone plasmid pGro7 (Takara, Tokyo, Japan). The expression vector for *Cc*Cel9A, *Cc*Cel9B, and BlgA had been constructed in our previous study (Zhang et al., 2014; Meng et al., 2015).

### Enzyme expression and purification

All genes were over-expressed in *E. coli* BL21(DE3) and the recombinant strains were incubated at 19°C overnight at 200 rpm. Except for that the expression of *cel48a* needed additional 4 mg ml^−1^ L-arabinosel, 500 mmol l^−1^ isopropyl β-D-1-thiogalactopyranoside (IPTG) was used to induce the cells at OD_600_ of 0.6. Cells were collected by centrifugation (7000 × g for 20 min at 4°C) after induction. The cell pellets were resuspended in lysis buffer (50 mmol l^−1^ NaH_2_PO_4_, 300 mmol l^−1^ NaCl, pH 8.0) and disrupted by ultrasonication after adding DNase and protease inhibitor. The target protein was purified by affinity chromatography using Ni-nitrilotriacetic acid (NTA) His-Bind resin according to the supplier's protocol (Novagen, Darmstadt, Germany). The purity was analyzed by SDS-PAGE and the purified protein was ultracentrifuged and resuspended in 50 mmol l^−1^ HAc-NaAc buffer (pH 5.0). Protein concentration was determined by using the Pierce BCA Protein Assay Kit (Thermo Fisher Scientific, Rockford, USA).

### Enzyme activity and processivity assay

For enzyme activity assay, 1 μmol l^−1^ of the purified enzymes were incubated with 20 mg ml^−1^ CMC for 30 min and 5 mg ml^−1^ filter paper for 4 h, respectively. All samples were prepared in 50 mmol l^−1^ HAc-NaAc buffer (pH 5.0) and the concentrations of soluble reducing sugars were measured using 3,5-dinitrosalicyclic acid method (Gusakov et al., 2011). The processivity was determined according to a motified protocol of Inci et al (Ozdemir et al., 2012). Single discs of filter paper with a diameter of 10 mm and a total weight of 3 mg were incubated with 9 μM purified enzymes at 60°C in 50 mmol l^−1^ HAc-NaAc buffer (pH 5.0) for 16 h. After incubation, the supernatant (150 μl) containing soluble reducing sugar was removed and the residual filter papers were washed with the same buffer for three times. After that, 150 μl of 50 mmol l^−1^ HAc-NaAc buffer was added to the filter paper. DNS reagent (200 μl) was added to the supernatant and the filter paper tubes for the measurement of the reducing sugars.

### Product pattern of the purified enzyme

The enzymatic hydrolysis product of soluble cello-oligosaccharide was analyzed by thin-layer chromatography (TLC). The sample was separated on a 60 F_254_ silica gel plate (Merck, Darmstadt, Germany) with a developing solution of butanol/acetate/H_2_O (2:1:1, v/v) and the sugar was detected by charring with an acid/alcohol solution of ethanol/sulfuric acid (4:1, v/v).

### Calculation of gray scale value (GSV)

To realize the approximate concentration ratios of Ccel_3880:Ccel_3883 (*Cc*Cel9A:*Cc*Cel9B), the gray scale values of their bands on the endoglucanase-zymogram were calculated using ImageJ 1.42q (http://rsbweb.nih.gov/ij/) as described in Zhang et al. (2015). According to the LC-MS identification results, each pixel region of the bands 14 and 15 were extracted and transformed to GSV and the total value was noted as GSV of *Cc*Cel9A. In the same way, the GSV of *Cc*Cel9B was obtained by analyzing the bands 17 and 18, finally calculating GSV_Cel9A_/GSV_Cel9B_ as the ratio.

### Assessment of synergy and product analysis

Enzyme mixtures including *Cc*Cel9A, *Cc*Cel9B, *Cc*Cel48A, and BlgA with a total concentration of 1 μmol l^−1^ were incubated with CMC or filter paper in 50 mmol l^−1^ HAc-NaAc buffer (pH 5.0) and the concentrations of soluble reducing sugars were measured as described above. The optimal ratio of each cocktail was determined according to the release of reducing sugars. All experiments were carried out in triplicate. In order to assess the synergy of each cocktail, the following formula to calculate the degree of synergy (DOS) was used: DOS = (total sugars released from enzyme mixture)/(total sugars released from incubation with individual enzymes) (Iakiviak et al., 2011).

The enzyme mixtures were incubated at 60°C with 5 mg ml^−1^ filter paper or 0.5 mg ml^−1^ of steam-exploded corn stalk and samples were withdrawn for further analysis at intervals. The products of the hydrolysate were analyzed by high-performance anion-exchange chromatography with pulsed amperometric detection (HPAEC-PAD) using Dionex ICS-3000 (Sunnyvate, USA).

### Statistical analysis

Kruskal Wallis H test was applied to ascertain significant differences using predictive analytic software (PASW 18.0). The level of statistical significance was *P* < 0.05.

## Results and discussion

**Figure 1:**
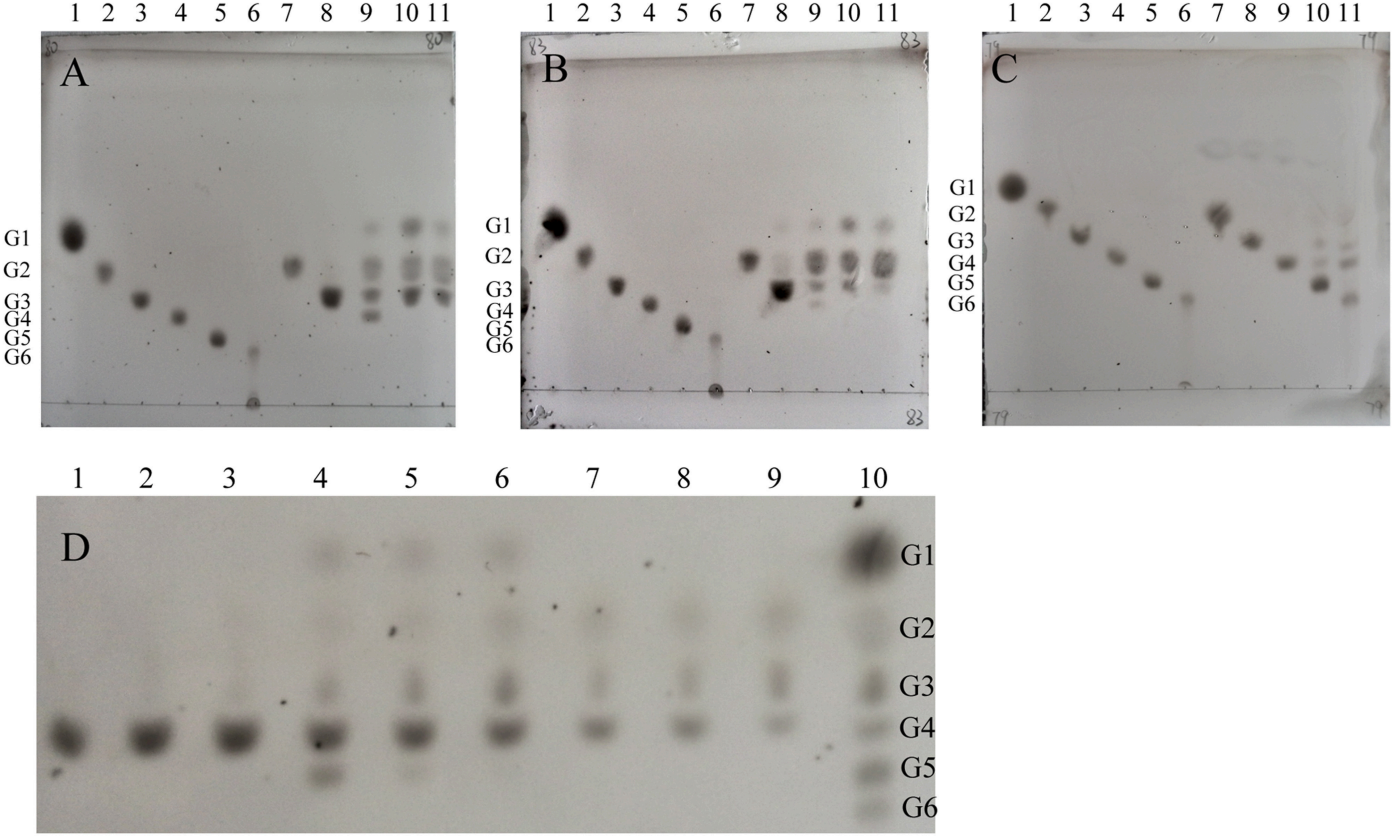
**Product patterns of *Cc*Cel9A(A,D), *Cc*Cel9B(B) and *Cc*Cel48A(C) on cello-oligosaccharides**. **(A–C)**: G1-G6 (lanes 1-6) refer to the positions of standards (4 μg of each): glucose (G1), cellobiose (G2), cellotriose (G3), cellotetraose (G4), cellopentaose (G5), and cellohexose (G6), respectively. Reactions containing G2-G6 (0.4%) were incubated at 60°C for 16 h with 0.5 μmol enzyme. Samples loaded in lanes 7-11 were the hydrolysis products of G2 (1 μL), G3 (2 μL), G4 (2 μL), G5 (2 μL), and G6 (3 μL), respectively. **(D)**: *Cc*Cel9A aliquots were incubated for 10, 30, and 120 min with cellotetraose (G4, lanes 1-3), cellopentaose (G5, lanes 4-6), and cellohexaose (G6, lanes 7-9). Lane 10, markers G1-G6 (from top down).

The molecular weights of *Cc*Cel9A, *Cc*Cel9B, *Cc*Cel48A, and BlgA were 115.8 kDa, 79.4 kDa, 101.5 kDa, and 54 kDa, respectively (ESM_1.pdf and ESM_2.pdf in Supplementary Material). Basic enzymatic characters (Table 1) showed that *Cc*Cel9A had both considerable endoglucanase and exoglucanase activities, with a remarkable processivity value. *Cc*Cel9B also performed efficiently on hydrolyzing soluble substrate, which showed as one of the major bands on the endoglucanase-zymogram. Although the specific activity of *Cc*Cel48A on filter paper was poor, it was the only active band identified on the exoglucanase-zymogram, suggesting that *Cc*Cel48A was the most significant exoglucanase in *C. cellulosi* CS-4-4 (Zhang et al., 2014). Hydrolysis of cello-oligosaccarides by these cellulases from *C. cellulosi* was determined by using TLC (Figure 1). The smallest substrates for *Cc*Cel9A and *Cc*Cel9B were both cellotetraose (G4). And they could hydrolyze cellopentaose (G5) and cellohexose (G6) to glucose (G1), cellobiose (G2), and cellotriose (G3) (Figures 1A,B). As shown in Figure 1D, G4 was released by the enzymatic hydrolysis of *Cc*Cel9A initially and then G4 were further broken down to G1, G2, and G3. However, the smallest substrate for *Cc*Cel48A was cellopentaose (G5), and it was able to cleave G5 and G6 to G2, G3, and G4 (Figure 1C). All of the cello-oligosaccharides including G2-G6 could be degraded to G1 completely by BlgA (Meng et al., 2015).

**Table 1:** **Enzymatic characters of *Cc*Cel9A, *Cc*Cel9B, and *Cc*Cel48A**.

**Enzyme**	**Specific activity**	**(U/μmol protein)**	**Processivity**
	**CMC**	**Filter paper**	
CcCel9A	678.8 ± 15.1	5.4 ± 0.39	11.14 ± 0.23
CcCel9B	869 ± 8.5	0.5 ± 0.06	1.02 ± 0.11
CcCel48A	ND	0.3 ± 0.02	3.65 ± 0.03

**Figure 2:**
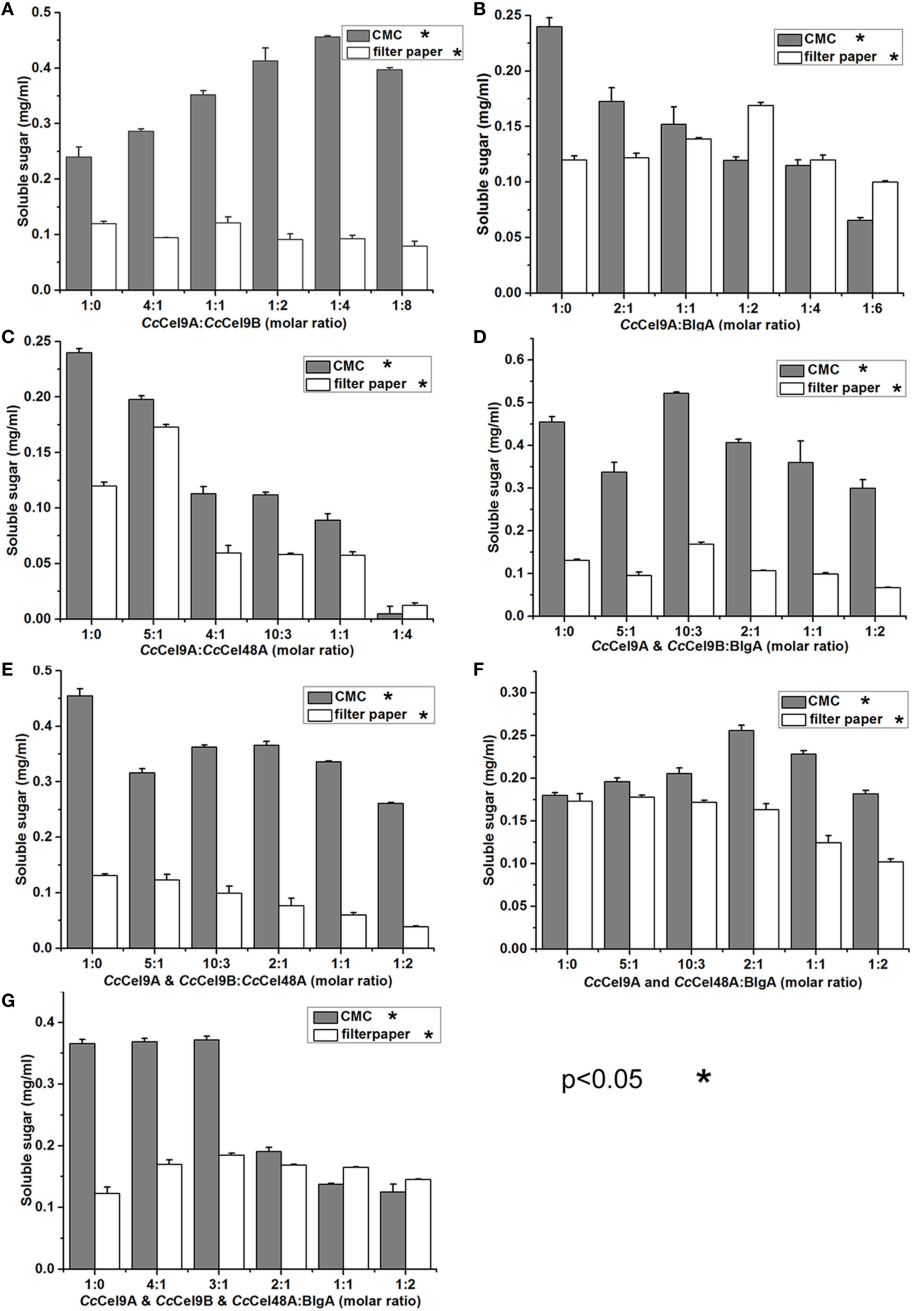
**Synergistic effects of a series of enzyme cocktails with a total concentration of 1 μmol l^−1^ incubated at 60°C with 20 mg ml^−1^ CMC for 30 min or 5 mg ml^−1^ filter paper for 4 h, respectively. (A)** The molar ratio of *Cc*Cel9A to *Cc*Cel9B were ranged from 1:0 to 1:8; **(B)** The molar ratios of *Cc*Cel9A to BlgA were ranged from 1:0 to 1:6; **(C)** The molar ratio of *Cc*Cel9A to *Cc*Cel48A were ranged from 1:0 to 1:4; **(D)** The molar ratios of the mixture (*Cc*Cel9A & *Cc*Cel9B) to BlgA were ranged from 1:0 to 1:2; **(E)** The molar ratios of the mixture (*Cc*Cel9A & *Cc*Cel9B) to *Cc*Cel48A were ranged from 1:0 to 1:2; **(F)** The molar ratios of the mixture (*Cc*Cel9A & *Cc*Cel48A) to BlgA were ranged from 1:0 to 1:2; **(G)** The molar ratios of the mixture (*Cc*Cel9A & *Cc*Cel9B & *Cc*Cel48A) to BlgA were ranged from 1:0 to 1:2.

The molar ratio of synergistic enzyme was crucial for efficient saccharification of cellulose (Kim et al., 2014). The approximate ratio of *Cc*Cel9A and *Cc*Cel9B on the endoglucanase-zymogram calculated by GSV method was 1:1, consequently the ratios of the purified *Cc*Cel9A and *Cc*Cel9B were optimized based on this data (Table 2). When *Cc*Cel9A was mixed with the non-processive endoglucanase *Cc*Cel9B, *Cc*Cel9B was better at hydrolyzing CMC while *Cc*Cel9A did better in filter paper (Figure 2A). BlgA complemented *Cc*Cel9A on both CMC and filter paper when they were mixed at 2:1 and 1:2, respectively (Figure 2B). The exoglucanase *Cc*Cel48A could stimulate the activity of *Cc*Cel9A on filter paper at a ratio of 5:1, but was redundant on hydrolyzing CMC (Figure 2C), which was consistent with the results described in the previous study (Zhang et al., 2010; Kostylev and Wilson, 2014). When *Cc*Cel9A, *Cc*Cel9B, and BlgA were mixed, the highest hydrolyzing efficiency on CMC was obtained (Figure 2D and Table 3). When *Cc*Cel9A, *Cc*Cel9B, and *Cc*Cel48A were mixed, weak synergy effect was observed (Figure 2E). In the mixture of *Cc*Cel9A, *Cc*Cel48A, and BlgA, the activity on filter paper was improved significantly (Figure 2F), whereas when all of the four enzymes were mixed together, the highest synergy effect on filter paper was achieved (Figure 2G).

**Table 2:** **The Gray scale value (GSV) of Ccel_3880 and Ccel_3883 on the endoglucanase-zymogram calculated by ImageJ**.

**Enzyme**	**Gray scale value**		**Total value**	**Ratio**
*Cc*Cel9A	3046.627(14)	3888.749(15)	6935.376	0.8723
*Cc*Cel9B	7560.033(17)	390.485(18)	7950.518	

**Table 3:** **The degree of synergy (DOS) for the enzyme cocktail hydrolyzing CMC and filter paper at the optimal ratio**.

**Enzyme cocktail**	**DOS (molar ratio)**	
	**CMC**	**Filter paper**
*Cc*Cel9A	1.0	1.0
*Cc*Cel9A : *Cc*Ce48A	0.9 (5:1)	1.7 (5:1)
*Cc*Cel9A : *Cc*Cel9B	1.1 (1:4)	1.1 (1:1)
*Cc*Cel9A : BlgA	1.0 (2:1)	2.1 (1:2)
*Cc*Cel9A & *Cc*Cel9B : *Cc*Cel48A	1.2 (2:1)	1.3 (5:1)
*Cc*Cel9A & *Cc*Cel9B : BlgA	1.9 (10:3)	1.5 (10:3)
*Cc*Cel9A & *Cc*Cel48A : BlgA	1.4 (2:1)	1.8 (5:1)
*Cc*Cel9A & *Cc*Cel9B & *Cc*Cel48A : BlgA	1.2 (3:1)	2.6 (3:1)

In the mixture, 1 μmol l^−1^ of enzyme cocktail was incubated at 60°C with 20 mg ml^−1^ CMC for 30 min or 5 mg ml^−1^ filter paper for 4 h, respectively.

**Figure 3:**
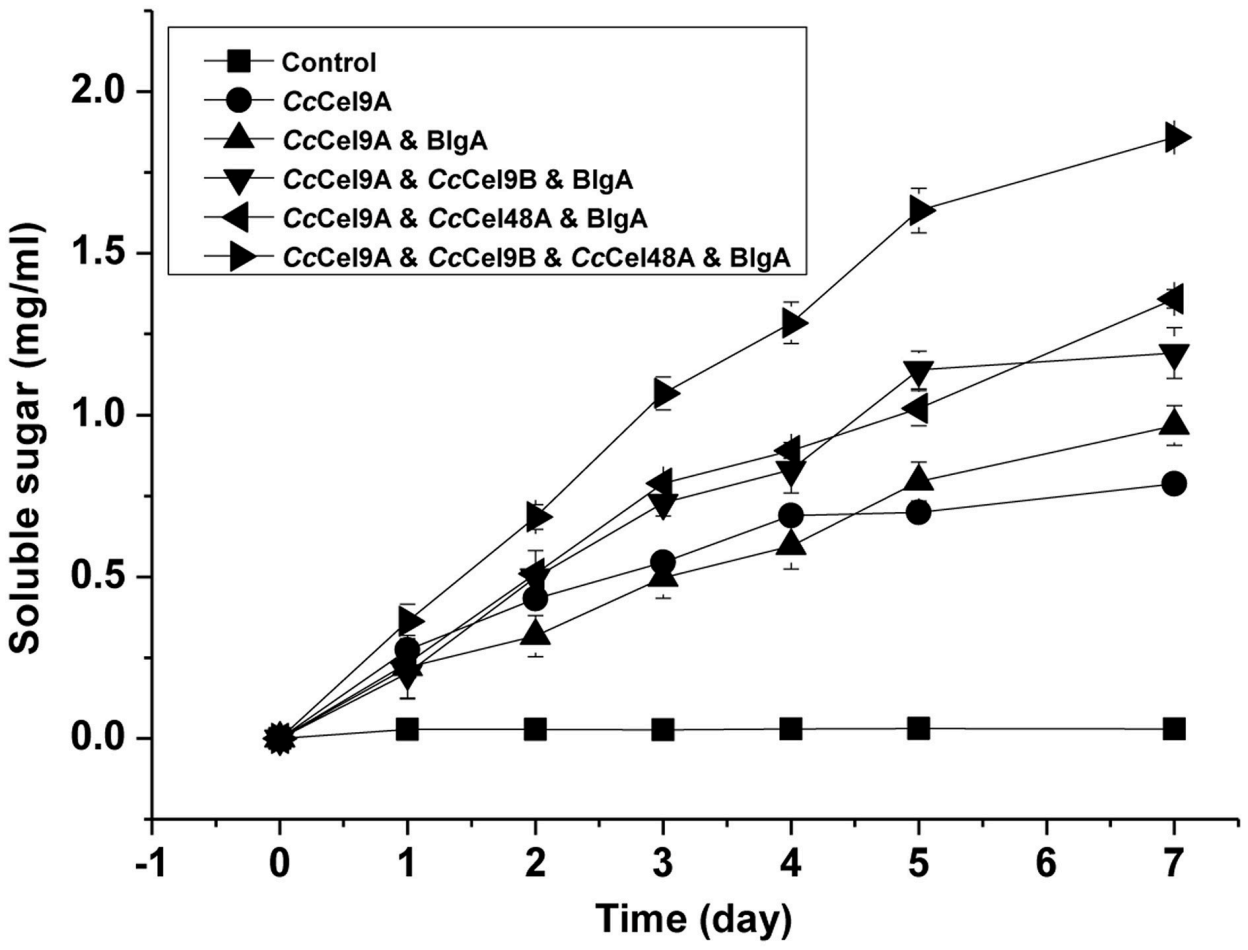
**Hydrolysis processes on filter paper of five enzyme cocktails**. Each cocktail with a total concentration of 1 μmol l^−1^ protein was incubated at 60°C with 5 mg ml^−1^ filter paper. The molar radios for each cocktail were set as follow: *Cc*Cel9A: BlgA = 2:1, *Cc*Cel9A: *Cc*Cel9B: BlgA = 5:5:3, *Cc*Cel9A: *Cc*Cel48A: BlgA = 25:5:6, and *Cc*Cel9A: *Cc*Cel9B: *Cc*Cel48A: BlgA = 25:25:10:18.

**Figure 4:**
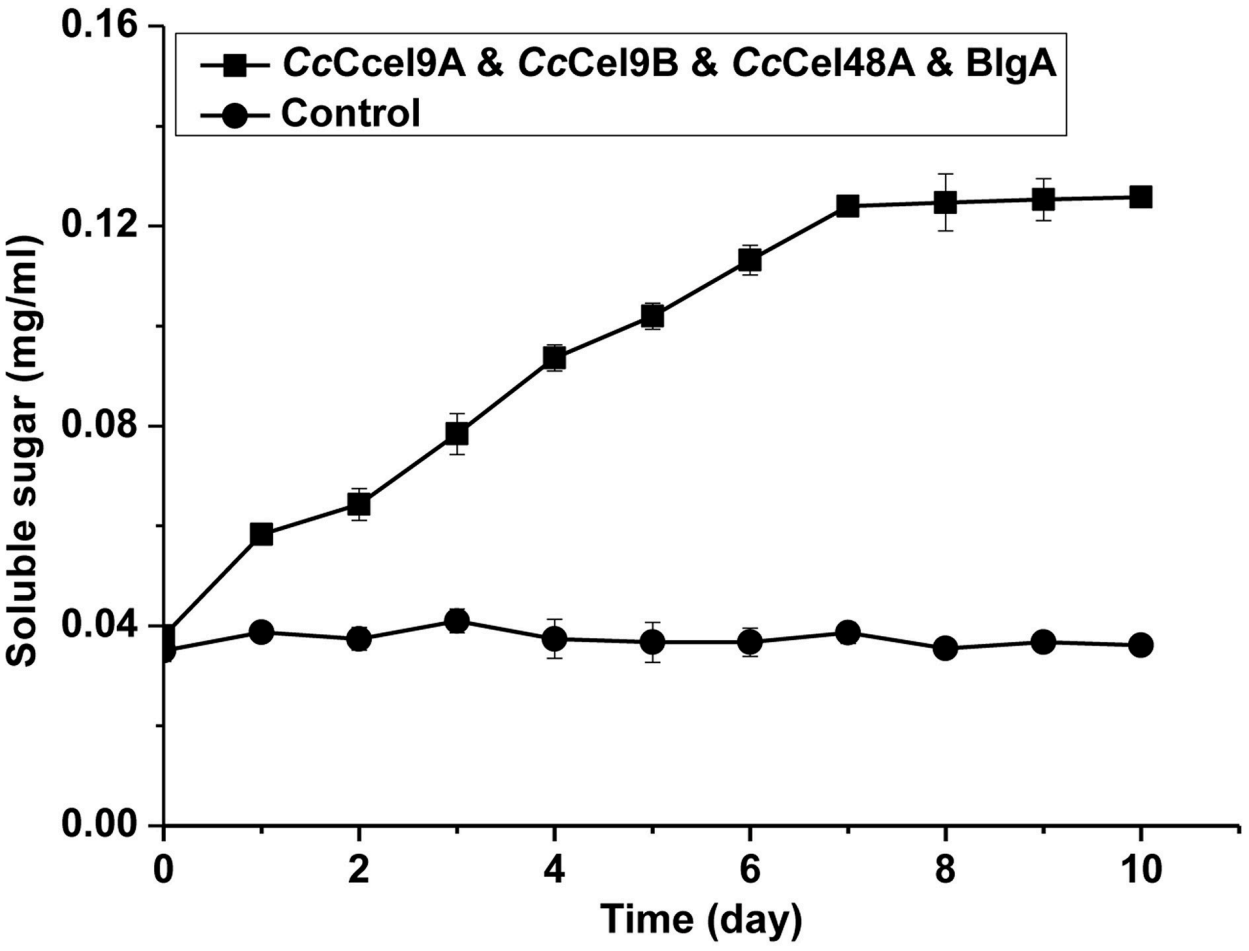
**Hydrolysis processes on corn stalk of the four-component enzyme cocktail (*Cc*Cel9A: *Cc*Cel9B: *Cc*Cel48A: BlgA = 25:25:10:18)**. Totally 1 μmol l^−1^ enzyme was incubated at 60°C with 0.5 mg ml^−1^ corn stalk.

The hydrolysis processes of filter paper by five enzyme cocktails were compared (Figure 3). The final concentrations of soluble sugars from *Cc*Cel9A, *Cc*Cel9A & BlgA, *Cc*Cel9A & *Cc*Cel9B & BlgA, *Cc*Cel9A & *Cc*Cel48A & BlgA, and *Cc*Cel9A & *Cc*Cel9B & *Cc*Cel48A & BlgA were 0.787, 0.968, 1.191, 1.359, and 1.859 mg ml^−1^, respectively. The hydrolysis rate of the four-component cocktail was highest with a sugar conversion rate of 33.5%, indicating the best synergy effect occurred among the multiple components of mixtures. Hence, it was applied to corn stalk hydrolysis and 15.8% of the substrate was converted to soluble sugars (Figure 4).

**Figure 5:**
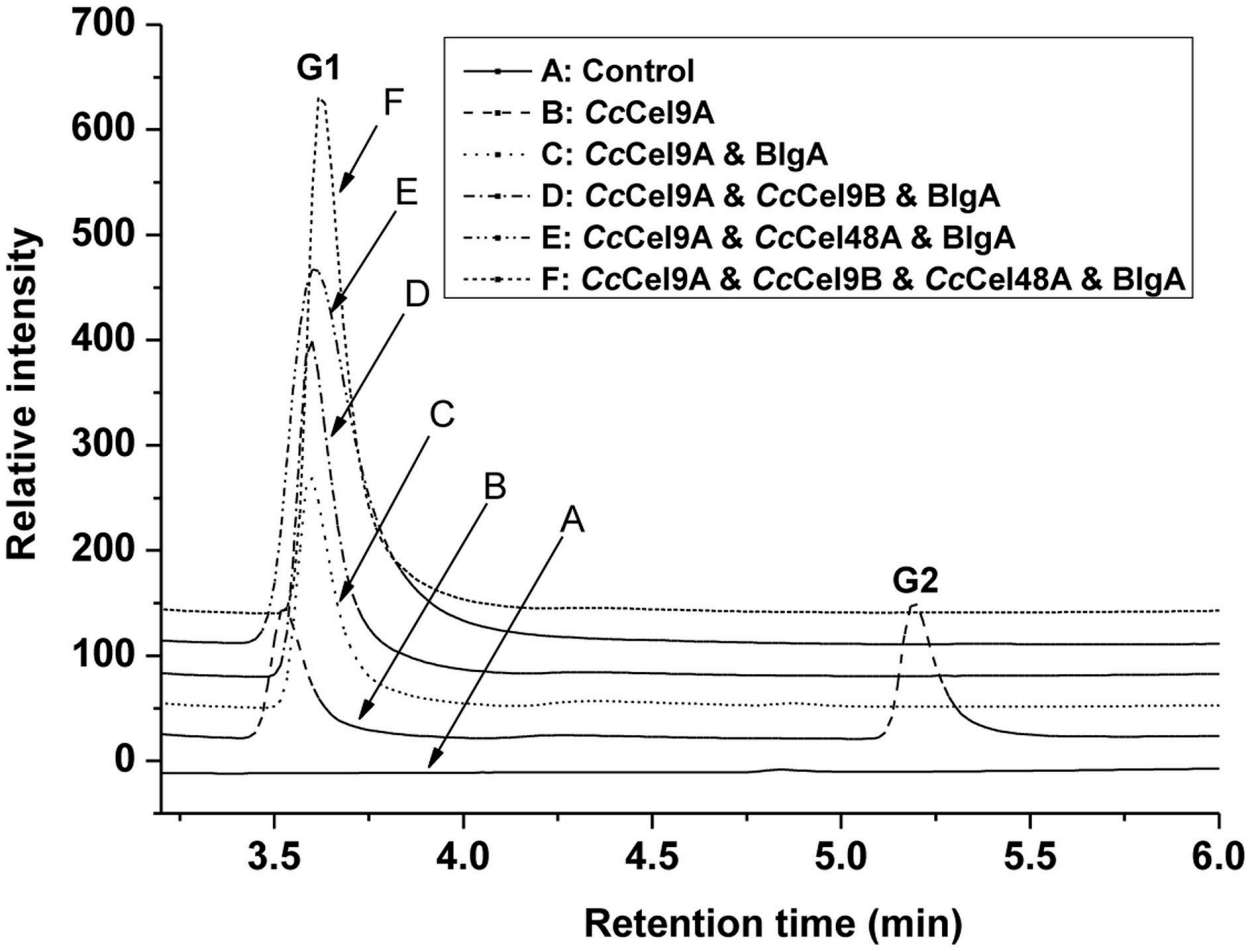
**Analysis of end products of filter paper hydrolyzed by enzyme cocktails using high-performance anion-exchange chromatography with pulsed amperometric detection**. Each cocktail with a total concentration of 1 μmol l^−1^ protein was incubated at 60°C with 5 mg ml^−1^ filter paper. The molar radios for each cocktail were set as follow: *Cc*Cel9A: BlgA = 2:1, *Cc*Cel9A: *Cc*Cel9B: BlgA = 5:5:3, *Cc*Cel9A: *Cc*Cel48A: BlgA = 25:5:6 and *Cc*Cel9A: *Cc*Cel9B: *Cc*Cel48A: BlgA = 25:25:10:18.

The products patterns of filter paper and corn stalk hydrolysis were detected by HPAEC-PAD after incubation at 60°C for a week (Figure 5 and ESM_3.pdf in Supplementary Material). Glucose was the only product of the mixtures, and the yield of glucose in the four-component cocktail was higher than the other cocktails. The mixture of 0.4 μM Cel9 and Cel48, from *C. phytofermentans*, was incubated with 10 mg ml^−1^ filter paper at 50°C for 72 h, and 1.620 mg ml^−1^ sugar containing G1, G2, G3, and G4 was obtained (Zhang et al., 2010). In our four-component cocktail, there was only glucose in the end product, which would be easily utilized by microorganisms for growth and fermentation.

**Figure 6:**
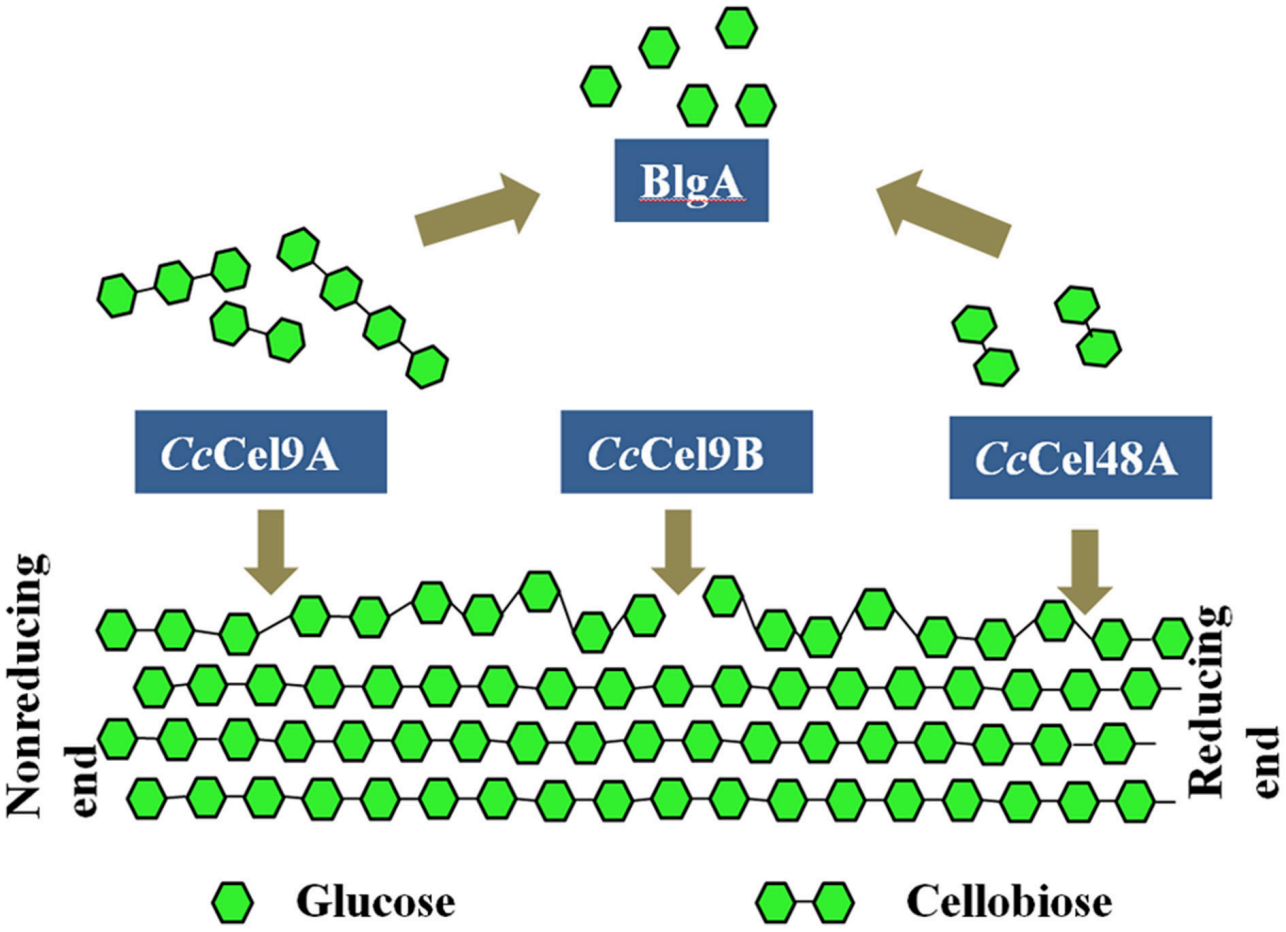
**Synergic deconstruction model of cellulose by *Cc*Cel9A, *Cc*Cel9B, *Cc*Cel48A, and BlgA**. *Cc*Cel9A and *Cc*Cel48A processively cleave cellulose from the end of a single chain, and oligosaccharides are released. *Cc*Cel9B, an endo-glucanase, cuts randomly in the cellulose chain. Oligosaccharides were hydrolyzed to glucose by BlgA.

Thermostable bacterial cellulase is the most popular candidate in various industries since it can work effectively under extreme conditions. In the present study, four different cellulases from two thermophilic microorganisms were mixed to obtain the optimal saccharification of cellulose (Figure 6). Therein, *Cc*Cel9A, *Cc*Cel9B, and *Cc*Cel48A were the major components in the secretome of *C. cellulosi* CS-4-4, which was isolated from decayed corn stalk with an optimal growth at 60°C (Zhang et al., 2014). *Cc*Cel9A, a multi-modular processive endoglucanase, could provide the four types of synergisms (Din et al., 1994; Teeri, 1997). On the soluble substrate CMC, *Cc*Cel9A, cooperating with the non-processive endoglucanase *Cc*Cel9B and BlgA, could hydrolyze cellulose to glucose. However, *Cc*Cel48A was unable to act on CMC, so there was no synergism after adding *Cc*Cel48A during the hydrolysis of CMC. On the crystalline substrate filter paper, *Cc*Cel48A could collaborate with *Cc*Cel9A to destroy the crystalline structure, synergizing with *Cc*Cel9A in the rate-limiting step (Figure 2 and Table 3). In addition, *Cc*Cel9A, *Cc*Cel9B, and *Cc*Cel48A are all multi-modular cellulases, synergism between the modules of them might occur in the hydrolysis process, which needed more detailed work.

## Conclusions

*Cc*Cel9A and *Cc*Cel48A from *C. cellulosi* CS-4-4 cooperate in destroying the crystalline structure of cellulose, providing single cellulose chain for endoglucanase *Cc*Cel9B to cut randomly. Meanwhile, *Cc*Cel9A and *Cc*Cel48A processively cleave cellulose from the end of a single chain and release G4 and G2, respectively. BlgA from *Caldicellulosiruptor* sp. F32 is capable of hydrolyzing G2-G6 to G1, which might remove the product inhibition for other cellulases (Kostylev et al., 2012). The ratios and cooperation of cellulases are crucial in the hydrolysis process and the efficiency could be further improved after adding some oxidoreductases (Gardner et al., 2014) or expansins (Georgelis et al., 2015), for which the bacterial enzyme pool is so abundant to excavate in the future.

## Author contributions

FL and KZ conceived and designed research; MY and KZ performed experiments and analyzed data; PZ and XM analyzed data; XZ provided technical assistance to KZ, XM, KZ, and FL wrote the manuscript; all authors commented on the manuscript and approved the contents.

## Funding

This work was supported by grants from the Shandong Province Natural Science Funds for Distinguished Young Scholar (No. JQ201507), the Key Scientific and Technological Project of Shandong Province (No. 2015ZDXX0403A01), and the Natural Science Foundation of China (No. 51561145015).

### Conflict of interest statement

The authors declare that the research was conducted in the absence of any commercial or financial relationships that could be construed as a potential conflict of interest.

## References

[B1] Arantes V. Saddler J. N. (2010). Access to cellulose limits the efficiency of enzymatic hydrolysis: the role of amorphogenesis. Biotechnol. Biofuels 3:4. 10.1186/1754-6834-3-4 20178562 PMC2844368

[B2] Berger E. Zhang D. Zverlov V. V. Schwarz W. H. (2007). Two noncellulosomal cellulases of *Clostridium thermocellum*, Cel9I and Cel48Y, hydrolyse crystalline cellulose synergistically. FEMS Microbiol. Lett. 268, 194–201. 10.1111/j.1574-6968.2006.00583.x 17227469

[B3] Din N. Damude H. G. Gilkes N. R. Miller R. C. Jr. Warren R. A. Kilburn D. G. (1994). C1-Cx revisited: intramolecular synergism in a cellulase. Proc. Natl. Acad. Sci. U.S.A. 91, 11383–11387. 10.1073/pnas.91.24.11383 7972069 PMC45235

[B4] Gardner J. G. Crouch L. Labourel A. Forsberg Z. Bukhman Y. V. Vaaje-Kolstad G. (2014). Systems biology defines the biological significance of redox-active proteins during cellulose degradation in an aerobic bacterium. Mol. Microbiol. 94, 1121–1133. 10.1111/mmi.12821 25294408

[B5] Georgelis N. Nikolaidis N. Cosgrove D. J. (2015). Bacterial expansins and related proteins from the world of microbes. Appl. Microbiol. Biotechnol. 99, 3807–3823. 10.1007/s00253-015-6534-0 25833181 PMC4427351

[B6] Gusakov A. V. Kondratyeva E. G. Sinitsyn A. P. (2011). Comparison of two methods for assaying reducing sugars in the determination of carbohydrase activities. Int. J. Anal. Chem. 2011:283658. 10.1155/2011/283658 21647284 PMC3103847

[B7] He Y. L. Ding Y. F. Long Y. Q. (1991). Two cellulolytic Clostridium species: *Clostridium cellulosi* sp. nov. and Clostridium cellulofermentans sp. nov. Int. J. Syst. Bacteriol. 41, 306–309. 10.1099/00207713-41-2-306 1854643

[B8] Iakiviak M. Mackie R. I. Cann I. K. (2011). Functional analyses of multiple lichenin-degrading enzymes from the rumen bacterium *Ruminococcus* albus 8. Appl. Environ. Microbiol. 77, 7541–7550. 10.1128/AEM.06088-11 21890664 PMC3209137

[B9] Jeoh T. Wilson D. B. Walker L. P. (2006). Effect of cellulase mole fraction and cellulose recalcitrance on synergism in cellulose hydrolysis and binding. Biotechnol. Prog. 22, 270–277. 10.1021/bp050266f 16454519

[B10] Jeon S. D. Yu K. O. Kim S. W. Han S. O. (2012). The processive endoglucanase EngZ is active in crystalline cellulose degradation as a cellulosomal subunit of *Clostridium cellulovorans*. N. Biotechnol. 29, 365–371. 10.1016/j.nbt.2011.06.008 21689799

[B11] Kim I. J. Lee H. J. Choi I. G. Kim K. H. (2014). Synergistic proteins for the enhanced enzymatic hydrolysis of cellulose by cellulase. Appl. Microbiol. Biotechnol. 98, 8469–8480. 10.1007/s00253-014-6001-3 25129610

[B12] Kostylev M. Wilson D. (2014). A distinct model of synergism between a processive endocellulase (TfCel9A) and an exocellulase (TfCel48A) from *Thermobifida fusca*. Appl. Environ. Microbiol. 80, 339–344. 10.1128/AEM.02706-13 24162578 PMC3910994

[B13] Kostylev M. Moran-Mirabal J. M. Walker L. P. Wilson D. B. (2012). Determination of the molecular states of the processive endocellulase *Thermobifida fusca* Cel9A during crystalline cellulose depolymerization. Biotechnol. Bioeng. 109, 295–299. 10.1002/bit.23299 21837665

[B14] Lynd L. R. van Zyl W. H. McBride J. E. Laser M. (2005). Consolidated bioprocessing of cellulosic biomass: an update. Curr. Opin. Biotechnol. 16, 577–583. 10.1016/j.copbio.2005.08.009 16154338

[B15] Mandelman D. Belaich A. Belaich J. P. Aghajari N. Driguez H. Haser R. (2003). X-ray crystal structure of the multidomain endoglucanase Cel9G from *Clostridium cellulolyticum* complexed with natural and synthetic cello-oligosaccharides. J. Bacteriol. 185, 4127–4135. 10.1128/JB.185.14.4127-4135.2003 12837787 PMC164890

[B16] Meng D. D. Ying Y. Zhang K. D. Lu M. Li F. L. (2015). Depiction of carbohydrate-active enzyme diversity in *Caldicellulosiruptor* sp. F32 at the genome level reveals insights into distinct polysaccharide degradation features. Mol. Biosyst. 11, 3164–3173. 10.1039/C5MB00409H 26392378

[B17] Ozdemir I. Blumer-Schuette S. E. Kelly R. M. (2012). S-layer homology domain proteins Csac_0678 and Csac_2722 are implicated in plant polysaccharide deconstruction by the extremely thermophilic bacterium *Caldicellulosiruptor saccharolyticus*. Appl. Environ. Microbiol. 78, 768–777. 10.1128/AEM.07031-11 22138994 PMC3264102

[B18] Riedel K. Bronnenmeier K. (1999). Active-site mutations which change the substrate specificity of the *Clostridium stercorarium* cellulase CelZ implications for synergism. Eur. J. Biochem. 262, 218–223. 10.1046/j.1432-1327.1999.00374.x 10231384

[B19] Sakon J. Irwin D. Wilson D. B. Karplus P. A. (1997). Structure and mechanism of endo/exocellulase E4 from *Thermomonospora fusca*. Nat. Struct. Biol. 4, 810–818. 10.1038/nsb1097-810 9334746

[B20] Teeri T. T. (1997). Crystalline cellulose degradation: new insight into the function of cellobiohydrolases. Trends Biotechnol. 15, 160–167. 10.1016/S0167-7799(97)01032-9

[B21] Zhang K. Chen X. Schwarz W. H. Li F. (2014). Synergism of glycoside hydrolase secretomes from two thermophilic bacteria cocultivated on lignocellulose. Appl. Environ. Microbiol. 80, 2592–2601. 10.1128/AEM.00295-14 24532065 PMC3993165

[B22] Zhang Q. Zhang X. Wang P. Li D. Chen G. Gao P.. (2015). Determination of the action modes of cellulases from hydrolytic profiles over a time course using fluorescence-assisted carbohydrate electrophoresis. Electrophoresis 36, 910–917. 10.1002/elps.201400563 25546561

[B23] Zhang X. Z. Sathitsuksanoh N. Zhang Y. H. (2010). Glycoside hydrolase family 9 processive endoglucanase from *Clostridium phytofermentans*: heterologous expression, characterization, and synergy with family 48 cellobiohydrolase. Bioresour. Technol. 101, 5534–5538. 10.1016/j.biortech.2010.01.152 20206499

[B24] Zverlov V. V. Schantz N. Schwarz W. H. (2005). A major new component in the cellulosome of *Clostridium thermocellum* is a processive endo-beta-1, 4-glucanase producing cellotetraose. FEMS Microbiol. Lett. 249, 353–358. 10.1016/j.femsle.2005.06.037 16006068

[B25] Zverlov V. V. Velikodvorskaya G. A. Schwarz W. H. (2003). Two new cellulosome components encoded downstream of celI in the genome of Clostridium thermocellum: the non-processive endoglucanase CelN and the possibly structural protein CseP. Microbiology 149(Pt 2), 515–524. 10.1099/mic.0.25959-0 12624213

