# Supplementary material for: Synergistic Cellulose Hydrolysis Dominated by a Multi-Modular Processive Endoglucanase from Clostridium cellulosi
Source: Front Microbiol. 2016 Jun 15;7:932. doi: 10.3389/fmicb.2016.00932 (PMC4908102; doi:10.3389/fmicb.2016.00932)

**ELECTRONIC SUPPLEMENTARY MATERIAL**

**Hydrolysis of Cellulose by a Multi-modular** **Processive Endoglucanase from** **Thermophilic Bacterium *Clostridium cellulosi***

**ESM_1.pdf** SDS-PAGE of the purified enzymes used in this study. Lane 1, protein molecular mass marker; lane 2, BlgA; lane 3, *Cc*Cel48A; lane 4, *Cc*Cel9B; lane 5, *Cc*Cel9A. Portions (2 µg) of each enzyme were analyzed on an 8% SDS-polyacrylamide gel.


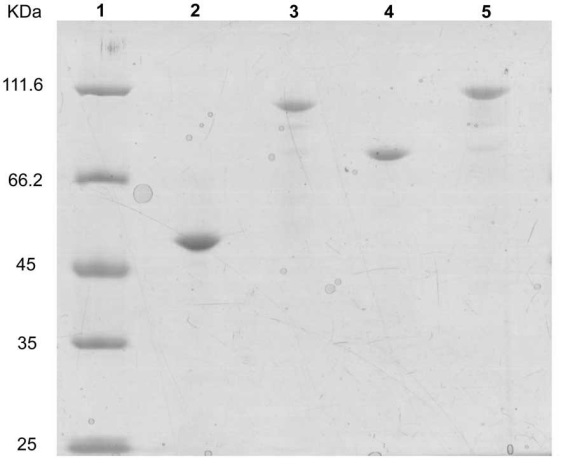


**ESM_2.pdf** Schematic structures of CcCel9A, CcCel9B, CcCel48A and BlgA (Meng et al. 2015; Zhang et al. 2014).


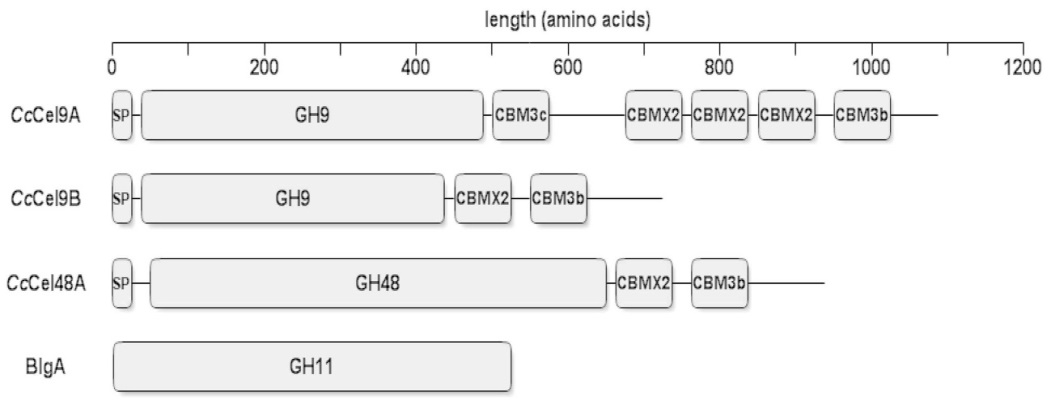


**ESM_3.pdf** Analysis of end products of corn stalk hydrolyzed by the four-component cocktail using high-performance anion-exchange chromatography with pulsed amperometric detection (HPAEC-PAD).


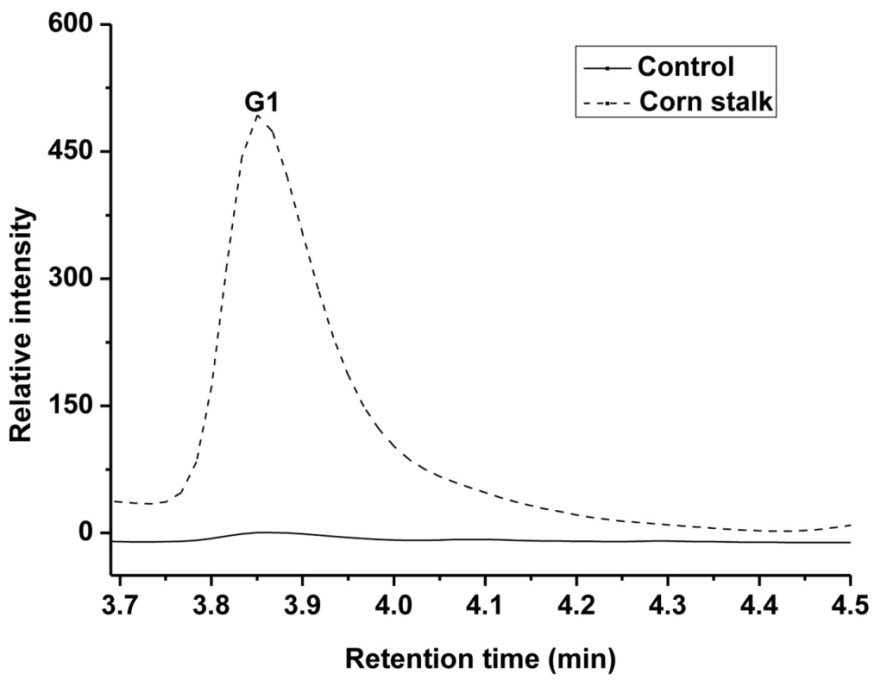

Supplement: Supplementary file 1 [file DataSheet1.docx]
